# Three-year follow-up of the COVAXID trial: real-world assessment of SARS-CoV-2 mRNA vaccine immunogenicity in immunocompromised individuals highlights increasing roles of hybrid and passive immunity

**DOI:** 10.1016/j.ebiom.2026.106279

**Published:** 2026-05-08

**Authors:** Puran Chen, Peter Bergman, Ola Blennow, Lotta Hansson, Stephan Mielke, Piotr Nowak, Yu Gao, Gunnar Söderdahl, Anders Österborg, C.I. Edvard Smith, Jan Vesterbacka, David Wullimann, Angelica Cuapio, Mira Akber, Gordana Bogdanovic, Sandra Muschiol, Mikael Åberg, Karin Loré, Margaret Sällberg Chen, Per Ljungman, Marcus Buggert, Soo Aleman, Hans-Gustaf Ljunggren

**Affiliations:** a Department of Medicine Huddinge, Center for Infectious Medicine, Karolinska Institutet, Stockholm, Sweden; b Department of Infectious Diseases, Karolinska University Hospital, Stockholm, Sweden; c Department of Laboratory Medicine, Clinical Immunology, Karolinska Institutet, Stockholm, Sweden; d Department of Clinical Immunology and Transfusion Medicine, Karolinska University Hospital, Stockholm, Sweden; e Department of Transplantation, Karolinska University Hospital, Stockholm, Sweden; f Department of Clinical Science, Intervention and Technology, Karolinska Institutet, Stockholm, Sweden; g Department of Hematology, Karolinska University Hospital, Stockholm, Sweden; h Department of Oncology-Pathology, Karolinska Institutet, Stockholm, Sweden; i Department of Cellular Therapy and Allogeneic Stem Cell Transplantation (CAST), Karolinska University Hospital Huddinge, Karolinska Comprehensive Cancer Center, Stockholm, Sweden; j Department of Laboratory Medicine, Biomolecular and Cellular Medicine, Karolinska Institutet, Stockholm, Sweden; k Department of Medicine Huddinge, Infectious Diseases, Karolinska Institutet, Stockholm, Sweden; l Department of Clinical Microbiology, Karolinska University Hospital, Stockholm, Sweden; m Department of Microbiology, Tumor and Cell Biology, Karolinska Institutet, Stockholm, Sweden; n Department of Medical Sciences, Clinical Chemistry, Science for Life Laboratory, Uppsala University, Uppsala, Sweden; o Department of Medicine Solna, Karolinska Institutet, Stockholm, Sweden; p Department of Laboratory Medicine, Division of Pathology, Karolinska Institutet, Stockholm, Sweden; q Department of Medicine Huddinge, Division of Hematology, Karolinska Institutet, Stockholm, Sweden

**Keywords:** SARS-CoV-2, COVID-19, mRNA vaccine, Clinical study, Primary immunodeficiency disease, HIV, Solid organ transplantation, Haematopoietic stem cell transplantation, Chronic lymphocytic leukaemia

## Abstract

**Background:**

Immunocompromised individuals were identified early in the pandemic as being at increased risk of severe COVID-19 and have demonstrated variable immune responses to SARS-CoV-2 vaccination. Although coordinated vaccination programmes are now well established, their long-term effects on sustained immunity in the present patient populations remain insufficiently understood.

**Methods:**

The prospective SARS-CoV-2 mRNA vaccine trial COVAXID was conducted in a well-characterised, real-world cohort of 539 immunocompromised and healthy individuals across 21 subgroups, organised into six main categories. At the 36-month time point, 218 participants remained. Participants provided blood samples for assessment of binding antibody titres and pseudo-neutralisation activity against ancestral SARS-CoV-2 and 21 variants, including Omicron sub-lineages. T cell responses were evaluated in a defined subset of participants. Immunogenicity outcomes were analysed over a three-year period in relation to SARS-CoV-2 vaccination, SARS-CoV-2 infection, and immunoglobulin replacement therapy (IGRT).

**Findings:**

Between years two and three, antibody titres and neutralisation capacity showed a consistent pattern of maintenance or increase across most study groups and subgroups. These increases were driven by cumulative exposure to vaccine booster doses, SARS-CoV-2 infection, and, in some cases, passive immunisation through IGRT. CD4^+^ and CD8^+^ T cell responses were detected across all study groups. Early immune responses were primarily vaccine-driven, whereas later immune profiles reflected substantial contributions from natural infection and anti-SARS-CoV-2 antibodies in IGRT products.

**Interpretation:**

The findings support continued, tailored vaccination strategies for elderly and immunocompromised individuals. Integrating immune monitoring with infection history and adjunctive therapies may help refine booster policies, optimise protection, and strengthen future vaccination programmes for high-risk populations.

**Funding:**

The present studies were supported by the European Research Council, Karolinska Institutet, Knut and Alice Wallenberg Foundation, Nordstjernan AB, Region Stockholm, and the Swedish Research Council.

**Research in context**

**Evidence before this study**

Immunocompromised individuals were largely excluded from the pivotal mRNA vaccine trials conducted in 2020, underscoring the need for dedicated prospective clinical studies in these patient groups to inform vaccination policies in both clinical and public health settings. The COVAXID study was launched in early 2021 to address this gap and has since provided important insights into vaccine-induced immunity among immunocompromised populations. Interim analysis at day 35, one-year, and two-years demonstrated that immunocompromised individuals initially developed variable immune responses following the first two doses of mRNA vaccination. Responses improved after the third and fourth doses but remained low in certain subgroups, particularly those receiving immunosuppressive therapy. By the second year, hybrid immunity resulting from vaccination and natural infection contributed to broader immune responses. The present report provides the final three-year update from the COVAXID clinical trial, offering longitudinal data on immune responses to repeated SARS-CoV-2 mRNA vaccination, natural infection, and immunoglobulin replacement therapies (IGRT). This represents one of the longest prospective studies of SARS-CoV-2 immunogenicity in immunocompromised populations to date. To assess the uniqueness of this work, we performed a PubMed search on November 11, 2025, using the terms (“SARS-CoV-2” OR “COVID-19”) AND (“immunocompromised” OR “immunodeficient”) AND (“vaccination”) AND (“mRNA”), limited to “Clinical Trials,” which yielded 15 results. None reported follow-up durations of up to three years. A similar search limited to “Clinical Studies” yielded 44 results, likewise none without follow-up data extending to three years.

**Added value of this study**

This study presents a comprehensive three-year follow-up of immunogenicity within the COVAXID clinical trial cohort. Through longitudinal assessment of both humoural and cellular immune responses, it offers unique insights into cumulative and dynamic effects of repeated SARS-CoV-2 mRNA vaccination, natural infection, and IGRT in the studied patient populations. By the end of the third year of follow-up, most participants had experienced SARS-CoV-2 infection, enabling an in-depth evaluation of hybrid immunity in this high-risk population. Notably, even patient subgroups previously characterised by weak vaccine responses demonstrated longitudinal increases in antibody titres and neutralising activity, reflecting the additive effects of infection and vaccine boosting. In selected subgroups, contributions from passively transferred antibodies via IGRT were also evident. Together, these findings highlight the evolving immune landscape in immunocompromised individuals and underscore the need for individualised vaccination strategies that account for infection history and IGRT exposure.

**Implications of all the available evidence**

The results highlight the interplay between vaccination, infection, and, in selected groups, IGRT in shaping SARS-CoV-2 immunity among immunocompromised patients. They underscore the continued need for tailored booster strategies that account for exposure history, immune status, and circulating variants. Updated vaccines adapted to prevailing SARS-CoV-2 strains remain crucial, particularly for immunocompromised and elderly populations. This and other long-term studies provide essential information to guide future public health strategies.

## Introduction

In January 2020, the World Health Organisation (WHO) declared Coronavirus Disease 2019 (COVID-19) a Public Health Emergency of International Concern (PHEIC) and subsequently classified it as a pandemic in March 2020.^1^^,^^2^ Early in the pandemic, immunocompromised individuals were identified as a high-risk group for severe COVID-19 and mortality,^3^ a pattern that persisted during the early Omicron period.^4^ Because the pivotal SARS-CoV-2 mRNA vaccine trials did not include immunocompromised populations,^5^^,^^6^ there was a clear need for prospective clinical studies to evaluate vaccine safety and immunogenicity in these individuals. To address this gap, the COVAXID clinical trial was initiated in March 2021, focussing on the anti-SARS-CoV-2 BNT162b2 mRNA vaccine in patients with primary or secondary immunodeficiencies.^7^

Initial data from the COVAXID clinical trial showed that two doses of the BNT162b2 mRNA vaccine were safe, although some adverse immune activation phenomena were observed. Two weeks after the second dose, antibody (Ab) responses varied widely across immunocompromised patient groups.^7^ Follow-up analyses at six and twelve months revealed heterogeneous increases in Spike IgG titres and neutralisation capacity against several early SARS-CoV-2 variants following three vaccine doses.^8^ However, neutralisation against early Omicron variants remained markedly low. A fourth vaccine dose, or a combination of three doses and natural infection, led to substantially improved Omicron-specific neutralisation in several immunocompromised subgroups. Despite these improvements, certain groups, notably those with specific primary immunodeficiencies or receiving immunosuppressive treatment, continued to exhibit weak serological responses.

By 18- and 24-month follow-up, many patient groups that had initially demonstrated poor responses to primary vaccination and early booster doses showed improved immunogenicity, reflected by higher Ab titres and enhanced neutralisation capacity.^9^ These responses correlated positively with the cumulative number of vaccine doses received and with prior SARS-CoV-2 infection. Nonetheless, underlying immunosuppression, whether disease-related or treatment-induced, continued to attenuate vaccine- and infection-induced responses in specific subgroups. Together, these findings underscored the importance of continued booster dosing to establish and maintain protective Ab responses in immunocompromised individuals.

The present study provides a comprehensive three-year (36-month) follow-up analysis of the COVAXID cohort, evaluating binding Ab titres, neutralisation capacity, and cellular responses against the ancestral SARS-CoV-2 strain and emerging Omicron variants. These extended analyses also consider the influence of additional booster doses, infection history, and immunoglobulin replacement therapy (IGRT) on immune responses. The results illustrate a transition from vaccine-induced to infection-driven (“hybrid”) immunity, with sustained increases in Abtitres and neutralising responses across nearly all study groups. Collectively, these findings demonstrate that repeated antigenic exposure, through SARS-CoV-2 vaccination and natural infection, can progressively strengthen immune responses even in intrinsically low responders, such as individuals with primary or secondary immunodeficiencies.

## Methods

### The COVAXID clinical trial

The prospective open-label clinical trial COVAXID has previously been described.^7^ The trial was registered in EudraCT (2021-000175-37) and ClinicalTrials.gov (NCT04780659). In short, inclusion criteria included individuals ≥18 years old with no known history of SARS-CoV-2 infection who had either primary or secondary immunodeficiency disorders (see below). Patients were recruited for the study during out-patient visits across various specialities at the Karolinska University Hospital, Stockholm, Sweden, with the selection process being impartial to sex. A healthy control (HC) group consisted of individuals without an immunocompromised disorder and/or immunomodulatory treatment. The original clinical trial protocol was set to conclude at 6 months after the second vaccine dose. It included two doses (days 0 and 21) of mRNA BNT162b2 (Pfizer/BioNTech) and immunogenicity measurements at six timepoints (days 0, 10, 21, 35, and months 3 and 6). After the initial six-months period, the cohort continued to be followed as part of an ongoing clinical study for up to three years, with subsequent analyses reported at 12 months (1 year),^8^ and 24 months (2 years).^9^ Blood samples and associated clinical data for the present three-year analysis were collected at 30 months (August 22nd, 2023, to October 30th, 2023) and 36 months (February 12th, 2023, to May 8th, 2024). The 30 months sampling time point was median 23.0 days from target day (range −17.6 to 45.4 days, CI 95%) and the 36-month sampling time point was median 19.0 days from target day (range −25.9 to 47.8, CI 95%). 232 study participants of the original 539 study participants provided blood samples for analyses at 30 and 36 months. Demographic data such as age and sex, and other medically relevant information were collected via electronic health records and a national vaccination register (Vaccinera). Subgroups were defined based on criteria set at the initiation of the clinical trial. The average follow-up time was 1097 days after the second vaccine dose (day 21). From a methodological standpoint, the blood draw process and the hospital-integrated biobank workflow were standardised over the entire three-year period. The Swedish COVID-19 vaccination recommendations during the study period are described in Supplementary Text S1.

### COVAXID study cohort

Patient groups with primary immunodeficies (PID), also termed inborn errors of immunity, included subgroups with common variable immunodeficiency (CVID), X-linked agammaglobulinemia (XLA), monogenic diseases, CD4-cytopenia, and other conditions. Patient groups with secondary immunodeficiency disorders included groups infected with human immunodeficiency virus (HIV) (including subgroups with ≤300 CD4 counts and >300 CD4 counts (cells/μl) at the initiation of the clinical trial); allogeneic haematopoietic stem cell transplantation (HSCT)/chimeric antigen receptor T (CAR T) cell therapy (including subgroups with HSCT within 6 months, 6–12 months, or >12 months at the initiation of the clinical trial); solid organ transplantation (SOT) (including subgroups <6 months with MMF, >6 months with MMF, and >6 months w/o MMF at the initiation of the clinical trial); and chronic lymphocytic leukaemia (CLL) (including subgroups with ibrutinib, off ibrutinib, indolent, and BR/FCR at the initiation of the clinical trial, where BR/FCR refers to “bendamustine and rituximab” and “fludarabine, cyclophosphamide and rituximab”). The control group consisted of individuals without an immunocompromised disorder or treatment, and without significant co-morbidity. The healthy controls were selected to represent three age groups (18–39 years, 40–59 years, and >60 years at the initiation of the clinical trial). For more details, see reference.^7^

### Procedures

Adding to previous collections at days 0, 10, 21, and 35, as well as at 3, 6, 9, 12, 18, and 24 months, serum, plasma and PBMC for the present analyses were collected at 30 and 36 months and analysed for anti-SARS-CoV-2 Ab titres and pseudo-neutralisation as well as T cell reactivity. The study subjects initially received monovalent mRNA vaccines according to label during the study period (predominantly BNT162b2/Comirnaty mRNA, Pfizer-BioNTech and in some cases mRNA-1273/Spikevax, Moderna). In the last two years of the study, many of the study participants obtained updated bivalent mRNA vaccines (BNT162b2 BA.1, BNT162b2 BA.4-5, and BNT162b2 XBB1.5, Pfizer-BioNTech) and (mRNA-1273 BA.1 and mRNA-1273 BA.4-5, Moderna). In rare cases (<1% of all provided vaccine doses), study subjects received protein vaccines (NVX-CoV2373, Novavax). Vaccine doses received by study subjects followed the recommendations of the Public Health Agency of Sweden. Clinical study-associated data were recorded in an electronic case report form (eCRF) as described. The presence of prior SARS-CoV-2 infection was recorded via in-person or phone interviews at each sampling time point, along with COVID-19 severity grading. Infections confirmed by PCR and/or rapid antigen test^7^ were accepted as a verified SARS-CoV-2 infection. Conducted PCR tests were verified through a manual review of the patients' electronic health records. Home-testing^7^ initiated at the patient's own initiative was recorded via in-person or phone interviews in connection with each sampling time point. Additionally, patients were retrospectively classified as having had a SARS-CoV-2 infection if IgG anti-nucleocapsid^1^ Ab titres were >5000 AU/mL (Meso Scale Discovery, MSD). Demographic data and vaccination records were completed for all analysed participants. Other clinical variables contained limited missing data (estimated to be <5% across essential variables) and were analysed using available-case approaches without imputation. Data on race or ethnicity were not collected due to local regulatory and ethical considerations, which should be considered when interpreting the generalisability of the findings.

### Ab tests

Serum samples from all study subjects included at 30 and 36 months were tested for IgG binding to SARS-CoV-2 Spike (JN.1), SARS-CoV-2 Spike (XBB.1.5), SARS-CoV-2 Spike (BA.2.86), SARS-CoV-2 Spike (BA.5), SARS-CoV-2 Spike (EG.5.1), SARS-CoV-2 N, SARS-CoV-2 S1 RBD, SARS-CoV-2 Spike, SARS-CoV-2 Spike (FL.1.5.1), SARS-CoV-2 Spike (JD.1.1), and SARS-CoV-2 Spike (HV.1) (Panel 38, Meso Scale Diagnostics, MSD). In addition, pseudo-neutralisation against SARS-CoV-2 Spike Wu-Hu.1 and the Omicron variants mentioned above was measured using the V-PLEX SARS-CoV-2 Panel 38 ACE2 kit (Meso Scale Diagnostics, MSD) from all study subjects included. All the above-mentioned analyses were performed at the SciLifeLab Affinity Proteomics Unit in Uppsala, Sweden. The assays were performed according to the manufacturer's instructions using a 1:50,000 dilution for the binding IgG assay and 1:100 dilution for the pseudo-neutralisation assay. Ab titres and neutralising capacity were expressed as arbitrary units (AU)/mL and % neutralisation, respectively. Additionally, serum samples were tested for pan-Ig, including IgG, to SARS-CoV-2 Wu-Hu.1 Spike receptor-binding domain (RBD) using the quantitative Elecsys anti-SARS-CoV-2 Spike enzyme immunoassay as described (Roche Diagnostics). Samples relying on the latter platform were analysed as per clinical routine, with additional dilutions if Ab titres were above the upper detection limit. Dilutions were performed to a maximum of 1:100.

### T cell tests

In a subgroup (n = 130; month 36) of all study subjects tested for Ab responses, PBMC were collected, and cellular samples were stimulated using peptide pools (15-mers with 11aa overlap; Peptide&Elephants) spanning the complete SARS-CoV-2 Spike glycoprotein from ancestral Wu-Hu.1 (WT) and Omicron variant BA.2.86. Briefly, lyophilised peptides were reconstituted at a stock concentration of 10 mg/mL in DMSO and diluted to 100 μg/mL in PBS. Cryopreserved PBMCs were thawed quickly, resuspended in complete medium in the presence of DNase I (10 U/mL; Sigma–Aldrich), and rested at 1 × 10^6^cells/well in 96-well U-bottom plates (Corning) for 3 h at 37 °C. For surface-stained analyses, the media was then supplemented with unconjugated anti-CD40 (clone HB14; Miltenyi) followed 15 min later by the relevant peptide pool (0.5 μg/mL). Cells were then incubated at 37 °C and 5% CO_2_ for 12 h. Negative control wells contained equivalent DMSO to the peptide pool. After stimulation, cells were washed in PBS supplemented with 2% FBS and 2 mM EDTA (FACS buffer). Cells were first stained for viability at room temperature, then CCR7 was added at 37 °C, followed by surface markers (CD3, CD4, CD8, CD14, CD19, CD45RA, CD69, CD137 and CD154) at room temperature. Cells were fixed in Cytofix fixation buffer (BD Biosciences) and acquired using a FACSymphony A5 (BD Biosciences). Data were analysed in FlowJo (version 10). Flow cytometry gating strategy shown in Supplementary Figure S1.

### Statistical analysis

Statistical analyses were performed using Python (version 3.10.1) with the SciPy statistics library (version 1.9.2). The study was designed as a prospective, real-world clinical cohort study, and analyses were primarily descriptive and exploratory in nature. Given the heterogeneity of the study population, small and uneven sample sizes across disease subgroups, and the presence of skewed distributions and outliers typical for immunological readouts, non-parametric statistical methods were selected a priori for all comparative analyses. Formal tests of normality were not used to guide test selection, as such tests have limited power and interpretability in small and unbalanced samples. Distributional properties of the data were assessed by visual inspection, which confirmed skewness and the presence of outliers across multiple variables. Non-parametric methods were therefore considered the most robust and conservative approach under these conditions. Between-group comparisons were performed using the Mann–Whitney U test for independent samples. For analyses involving more than two groups, appropriate non-parametric multiple-comparison approaches were applied, with Bonferroni correction used to control for type I error where applicable. Longitudinal comparisons across time points were evaluated within groups using non-parametric paired tests when relevant. Correlations between Ab binding titres and pseudo-neutralisation responses were assessed using Spearman's rank correlation coefficient. Antibody titres are presented as geometric means unless otherwise stated, while other summary statistics are reported as medians with interquartile ranges or 95% confidence intervals, as appropriate. Missing data were not imputed; all analyses were conducted using available-case data. Statistical significance was defined as a two-sided p value < 0.05. Exact statistical tests and sample sizes are specified in the respective figure legends.

### Ethical considerations

The clinical trial was approved by the Swedish Ethical Review Board and the Swedish Medical Products Agency (no. 2021-06046-02 and no. 5.1-2021-92151, respectively). Extension of the study was approved by the same bodies. Informed consent was obtained from all study participants prior to inclusion in the clinical trial and, additionally, before participating in the extension of the study.

### Role of funding source

The funders did not influence the study design, data collection, data analyses, interpretation, writing of the report, or decision to submit the paper for publication.

## Results

**Fig. 1:**
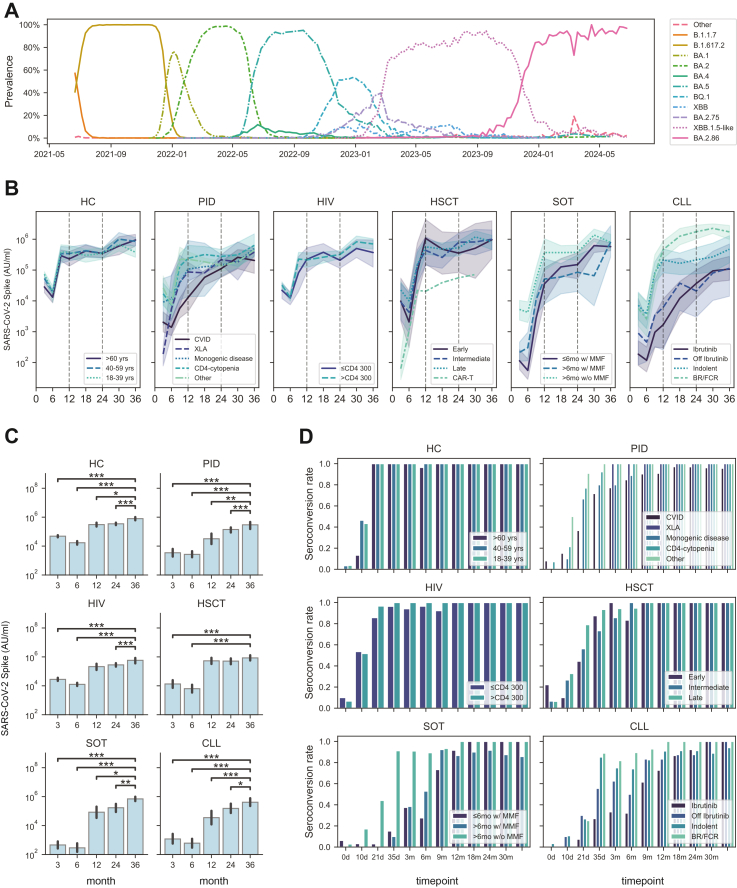
**Dynamics of SARS-CoV-2 antibody titres in the COVAXID cohort.** (A) Prevalence of major SARS-CoV-2 subvariants in Sweden during the 36-months study period. (B) Dynamics of Spike Wu-Hu.1 (WT) Ab titres (geometric mean with 95% CI, shaded range) for each study group and respective subgroups. The vertical dotted lines mark the 1-year and 2-year follow-up timepoints. (C) Bar plots of Spike Wu-Hu.1 Ab titres at 3, 6, 12, 24, and 36 months at the study group level. Statistical tests were performed using Mann–Whitney, and Bonferroni correction for multiple comparisons, using the 36-month time point as reference. (D) Seroconversion rates over time in each subgroup, defined as Spike receptor binding domain (RBD) titres ≥0.8 AU/mL. The star annotation (∗) indicates statistical significance at a p-value threshold of 0.05 (or ∗∗ for p < 0.01, ∗∗∗ for p < 0.001, ∗∗∗∗ for p < 0.0001).

In a real-world setting, we assessed SARS-CoV-2 immunogenicity in immunocompromised participants and healthy controls (HCs) from the original COVAXID cohort over a total period of three years. A detailed description of study groups and subgroups is provided in Table 1, which also summarises participant numbers at each timepoint up to 36 months, mean age at inclusion, proportion of females, average number of vaccine doses per year (0–12 months, 12–24 months, 24–36 months), recipients of bivalent vaccine doses, IGRT status, and SARS-CoV-2 infection history. During the 36-month study period, 11 major SARS-CoV-2 variants emerged that each reached a prevalence exceeding 10% at some point in Sweden (Fig. 1A).^10^ In the present analysis, particular emphasis was placed on immune responses measured between years two and three (24–36 months), as these data have not previously been reported. Between 24 and 30 months, the XBB.1.5 Omicron variant predominated, whereas between 30 and 36 months, BA.2.86 was the dominant circulating strain (Fig. 1A).

**Table 1:** Study cohort characteristics.

Study group	Subgroup defined at inclusion	Study participants at month 36 (n)	Average age at inclusion (years, range)	Proportion females	Average vaccine doses (n, range)				≥1 bivalent vaccine dose (n, %)	Number of patients with IGRT (n, %)	Proportion of infected individuals
					Month 0 to 12	Month 12 to 24	Month 24 to 36	Entire study period			
HC	>60 yrs	13	71 (62–79)	62%	3.1 (3–4)	1.7 (0–2)	0.8 (0–2)	5.4 (4–7)	10 (77%)	0 (0%)	92%
	40–59 yrs	15	55 (45–59)	53%	2.9 (2–3)	1.0 (0–1)	0.4 (0–2)	3.7 (2–5)	7 (47%)	0 (0%)	93%
	18–39 yrs	5	30 (25–35)	40%	3.0 (3–3)	1.0 (0–1)	0.0 (0–0)	3.2 (3–4)	1 (20%)	0 (0%)	100%
	**Total**	**33**	**57 (25–79)**	**55%**	**3.0 (2–4)**	**1.4 (0–2)**	**0.5 (0–2)**	**4.3 (2–7)**	**18 (55%)**	**0 (0%)**	**94%**
PID	CVID	27	56 (22–79)	63%	3.3 (2–4)	1.6 (0–2)	0.6 (0–2)	5.2 (2–8)	17 (63%)	25 (93%)	93%
	XLA	2	43 (39–47)	0%	3.5 (3–4)	2.0 (2–2)	0.5 (0–1)	6.0 (5–7)	1 (50%)	2 (100%)	100%
	Monogenic disease	2	34 (30–38)	50%	2.5 (2–3)	0.0 (0–0)	0.0 (0–0)	2.5 (2–3)	0 (0%)	0 (0%)	100%
	CD4-cytopenia	9	56 (29–78)	78%	3.3 (1–4)	1.3 (0–2)	0.3 (0–2)	4.7 (1–7)	4 (44%)	2 (22%)	78%
	Other	6	51 (22–68)	100%	2.7 (2–3)	1.4 (0–2)	0.5 (0–2)	4.3 (2–6)	4 (67%)	0 (0%)	67%
	**Total**	**46**	**54 (22–79)**	**67%**	**3.2 (1–4)**	**1.5 (0–2)**	**0.5 (0–2)**	**4.9 (1–8)**	**26 (57%)**	**29 (63%)**	**87%**
HIV	≤CD4 300	11	56 (34–71)	27%	2.7 (2–3)	1.3 (0–2)	0.6 (0–2)	4.0 (2–6)	7 (64%)	0 (0%)	64%
	>CD4 300	25	58 (33–76)	48%	2.8 (2–3)	1.5 (0–2)	0.6 (0–1)	4.3 (2–6)	15 (60%)	0 (0%)	88%
	**Total**	**36**	**58 (33–76)**	**42%**	**2.8 (2–3)**	**1.5 (0–2)**	**0.6 (0–2)**	**4.2 (2–6)**	**22 (61%)**	**0 (0%)**	**81%**
HSCT	Early <6 mo	4	60 (53–68)	50%	3.0 (1–4)	1.7 (0–2)	0.8 (0–1)	5.0 (1–7)	3 (75%)	1 (25%)	100%
	Intermediate 6–12 mo	5	56 (30–72)	40%	3.8 (3–4)	1.4 (1–2)	1.0 (1–1)	6.2 (5–7)	5 (100%)	1 (20%)	100%
	Late >12 mo	27	61 (34–74)	56%	3.7 (3–4)	1.6 (0–2)	0.9 (0–2)	5.9 (3–8)	23 (85%)	7 (26%)	85%
	CAR-T	0	–	–	–	–	–	–	–	–	–
	**Total**	**36**	**60 (30–74)**	**53%**	**3.6 (1–4)**	**1.6 (0–2)**	**0.9 (0–2)**	**5.8 (1–8)**	**31 (86%)**	**9 (25%)**	**89%**
SOT	≤6mo w/ MMF	10	51 (30–67)	50%	3.9 (3–4)	1.6 (0–2)	0.7 (0–2)	6.0 (3–8)	8 (80%)	0 (0%)	70%
	>6mo w/ MMF	8	59 (40–76)	62%	3.6 (3–4)	1.6 (0–3)	1.1 (1–2)	6.1 (5–8)	8 (100%)	0 (0%)	62%
	>6mo w/o MMF	9	57 (30–75)	67%	3.8 (3–4)	1.3 (0–2)	0.7 (0–2)	5.4 (4–7)	6 (67%)	0 (0%)	78%
	**Total**	**27**	**55 (30–76)**	**59%**	**3.8 (3–4)**	**1.5 (0–3)**	**0.8 (0–2)**	**5.9 (3–8)**	**22 (81%)**	**0 (0%)**	**70%**
CLL	Ibrutinib	8	72 (55–87)	25%	3.6 (3–4)	1.9 (1–3)	0.9 (0–2)	6.4 (4–8)	6 (75%)	4 (50%)	100%
	Off Ibrutinib	4	74 (66–86)	75%	3.5 (3–4)	1.8 (1–2)	0.8 (0–2)	6.0 (5–7)	3 (75%)	1 (25%)	75%
	Indolent	14	71 (49–82)	71%	3.5 (3–4)	1.5 (0–2)	0.6 (0–1)	5.4 (3–7)	10 (71%)	0 (0%)	71%
	BR/FCR	14	72 (57–84)	7%	3.6 (3–5)	1.6 (1–3)	0.9 (0–2)	6.2 (5–7)	13 (93%)	4 (29%)	93%
	**Total**	**40**	**72 (49–87)**	**40%**	**3.6 (3–5)**	**1.7 (0–3)**	**0.8 (0–2)**	**6.0 (3–8)**	**32 (80%)**	**9 (23%)**	**85%**

HC, Healthy controls; PID, Primary immunodeficiency; HIV, Human immunodeficiency virus; HSCT, Haematopoietic stem cell transplantation; SOT, Solid organ transplantation; CLL, Chronic lymphocytic leukaemia; CVID, Common variable immune deficiency; XLA, X-linked-agammaglobulinaemia; CAR-T, Chimeric antigen receptor T-cell; MMF, Mycophenolate mofetil; BR/FCR, Bendamustine and rituximab/fludarabine, cyclophosphamide and rituximab; IGRT, Immunoglobulin replacement therapy. Bold text represents the aggregated data for each respective study group.

>5000 AU/mL at any time during study period. Data represent all patients who provided a sample at the 36-month timepoint.

### SARS-CoV-2 binding Ab titres

Building on earlier assessments up to 24 months,^8^^,^^9^ we quantified binding Ab titres against the ancestral SARS-CoV-2 Wu-Hu.1 (WT) strain using the V-PLEX SARS-CoV-2 platform at 30 and 36 months (see Methods). Across all study groups and most subgroups, Ab titres showed a consistent pattern of maintenance or continued increase between 24 and 36 months (Fig. 1B and C; Supplementary Figure S2 and S3). This increase was particularly pronounced between months 24 and 30, coinciding with the emergence of the XBB.1.5 Omicron variant (Fig. 1B and C). Comparable results were obtained using the Elecsys clinical diagnostic platform (Supplementary Figure S4; compare with Fig. 1B), which was originally used to determine seroconversion, the primary endpoint of the COVAXID clinical trial (>0.8 AU/mL at day 35).^7^ Three years after trial initiation, all participants in the HC, HIV, and HSCT groups had seroconverted, either at this time point or earlier (Fig. 1D). At 36 months, a small subset of participants remained complete serological non-responders according to the protocol-defined seroconversion criteria used in the original COVAXID trial. These individuals were restricted to select immunocompromised subgroups, primarily those with profound immune deficiency or intensive immunosuppressive treatment (Fig. 1D).

### SARS-CoV-2 pseudo-neutralisation responses

**Fig. 2:**
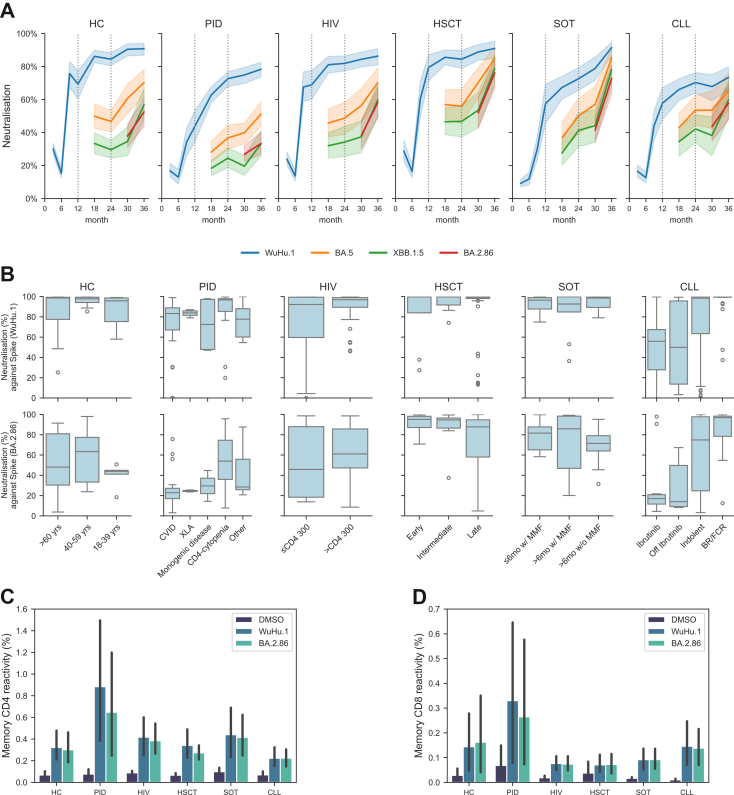
**Pseudo-neutralising SARS-CoV-2 antibody responses and cellular immunity.** (A) Dynamics of pseudo-neutralising responses against Spike Wu-Hu.1 (WT), BA.5, XBB1.5, and BA.2.82 (geometric mean with 95% CI, shaded range) for each study group. Vertical dotted lines mark the 1-year and 2-year follow-up points. (B) Box plots of pseudo-neutralising responses to Spike Wu-Hu.1 and BA.2.86 at 36 months across all study subgroups. (C) Bar plots showing percent responding CD4^+^ T cells against Wu-Hu.1 and BA.2.86 at the 36-month time point. (D) Bar plots showing percent responding CD8^+^ T cells against Wu-Hu.1 and BA.2.86 at the 36-month time point, calculated as a described for CD4^+^ T cells (median with 95% CI). For T cell analysis, HC (n = 14), PID (n = 13), HIV (n = 22), HSCT (n = 36), SOT (n = 14), and CLL (n = 31).

Earlier COVAXID analyses demonstrated that, although binding Ab titres were relatively stable across SARS-CoV-2 variants, pseudo-neutralising Ab responses were significantly lower against Omicron subvariants compared with WT and pre-Omicron variants. In the present study, we extended the analyses to include Omicron variants predominating during the 24-36-month period across the entire cohort. We first evaluated the correlation between Ab titres against WT and Omicron subvariants. Recent Omicron subvariants showed a high degree of correlation with earlier Omicron variants and the WT strain in terms of binding Ab titres at the 36-month time point (Supplementary Figure S5). In contrast, pseudo-neutralising Ab responses exhibited greater variability across Omicron subvariants. When pseudo-neutralisation responses against the predominant variants XBB1.5 and BA.2.86 were assessed, all major study groups demonstrated an overall longitudinal increase in neutralising capacity, reaching peak levels at 36 months (Fig. 2A). Within subgroups, the lowest responses were observed in CVID and XLA (PID) as well as in both the on- and off-ibrutinib subgroups (CLL), particularly against BA.2.86 (Fig. 2B). Notably, pseudo-neutralising responses in the HSCT group exceeded those observed in HCs (Fig. 2B).

### Cellular immune responses

Following serological analysis, we evaluated cellular immunity by assessing the percentage of responding cells against WT and the BA.2.86 Omicron variant at the 36-month time point (Fig. 2C and D). Antigen-specific CD4+ T cell responses were detected across all study groups (Fig. 2C). No significant differences were observed between patient groups and HCs, nor between responses to WT and BA.2.86 within the same groups (Fig. 2C). CD8+ T cell responses were also detected, although at lower levels, and followed similar patterns (Fig. 2D). No significant differences were observed between patient groups and HCs for either variant.

### Vaccination and infection frequencies

**Fig. 3:**
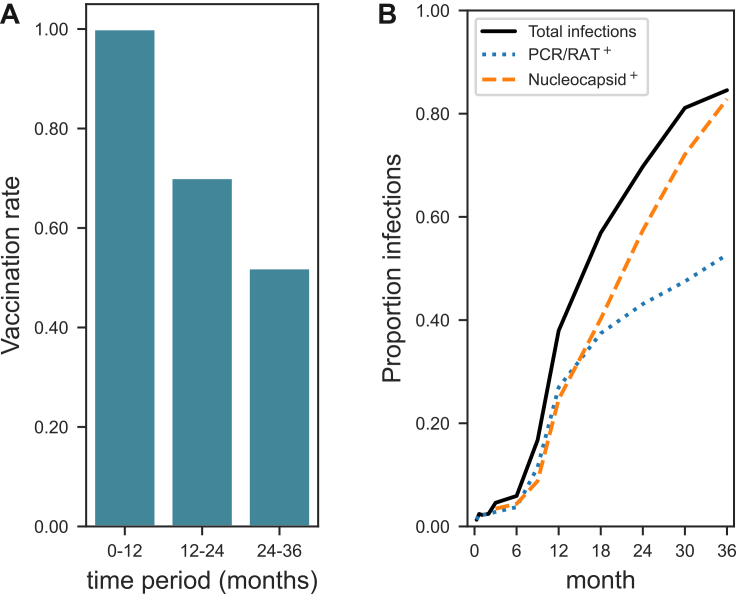
**SARS-CoV-2 vaccination and infection frequencies.** (A) Vaccination frequences over time, show as rate of study participants with one or more vaccination during the 0 to 12-, 12 to 24-, and 24 to 36-month periods across the entire cohort. (B) Infection frequencies over the 36 months period. Infections were assessed either by PCR/protein analysis or participant self-reporting (blue dotted line), and by detection of N-specific Abs in serum at sampling (orange dashed line). The combined frequency of infection detected by either method is shown in black (solid line).

Following the initial SARS-CoV-2 mRNA vaccine doses administered on days 1 and 21, participants continued to receive booster doses according to recommendations from the Public Health Agency of Sweden (Supplementary Text S1). As expected, vaccination frequency gradually declined over time across the cohort, reaching its lowest level during the 24-36-month period (Fig. 3A and Table 1). This decline coincided with the WHO's declaration on May 5, 2023, that COVID-19 was no longer a Public Health Emergency of International Concern. At study entry, all participants were SARS-CoV-2 naïve, consistent with the original trial's inclusion criteria. Over time, and particularly during the early Omicron wave, a substantial proportion of participants acquired natural infection. By 36 months, more than 80% had experienced at least one SARS-CoV-2 infection (Fig. 3B, Table 1). Whereas early Ab peaks corresponded to vaccine booster administration, later increases coincided with rising nucleocapsid (N) Ab levels, a correlate of natural infection. This pattern supports the interpretation that, beyond the second year of follow-up, SARS-CoV-2 infection became a major driver of humoural immunity within the cohort, complementing and, in some cases, surpassing the contribution of vaccine-induced responses (Supplementary Figure 6). Together, these findings indicate a gradual shift from vaccine-induced to infection-driven (“hybrid”) immunity within the cohort. In addition, passive Ab transfer through IGRT contributed to overall immunity in treated participants. Collectively, these results underscore the dynamic interplay between vaccination, natural infection, IGRT, and public health policies in shaping long-term immunity in immunocompromised and healthy populations.

## Discussion

This three-year follow-up of the COVAXID clinical trial provides a comprehensive long-term assessment of SARS-CoV-2 immunity in immunocompromised and healthy individuals. Spanning multiple variant waves, vaccine iterations, and public health recommendations, the data set offers real-world insights into how repeated antigenic exposure, through vaccination and infection, shapes immune responses in clinically vulnerable populations. With more than 80% of participants now having experienced SARS-CoV-2 infection in addition to multiple vaccine doses, the study captures the transition from vaccine-induced to infection-driven “hybrid” immunity.

A key finding from this extended follow-up is the consistent maintenance or increase of Ab titres and neutralising responses across nearly all immunocompromised subgroups and HCs between the 24- and 36-month assessments. This outcome contrasts with early concerns that such patients might fail to develop, or to sustain, durable immunity. While earlier COVAXID analyses at 35 days,^7^ one year,^8^ and two years^9^ demonstrated variable responsiveness depending on disease category and treatment, the present study shows that cumulative antigenic exposure, via booster vaccinations or natural infection, can progressively enhance immune function, even in groups with initially weak vaccine responses. The weaker correlation between binding Ab responses to recent Omicron variants (after BA.5) and the ancestral Wuhan strain likely reflects increasing antigenic divergence of Spike proteins, resulting in more variant-specific Ab responses with reduced cross-reactivity to ancestral antigens.

The high rate of SARS-CoV-2 infection in the third year underscores the major role of natural infection in shaping long-term immunity. Consistent with other studies demonstrating the superior breadth and magnitude of hybrid immunity compared with vaccination alone,^11^, ^12^, ^13^ our findings show enhanced neutralising capacity not only against early Omicron lineages (BA.1 and BA.2) but also against more antigenically divergent variants (XBB.1.5 and BA.2.86). The parallel rise in anti-N Abs and improved cross-neutralisation suggests that infection-driven exposure was a dominant contributor to late immunogenicity gains.

In addition to endogenous immunity, IGRT contributed to measured Ab levels in specific patient groups, particularly those with primary immunodeficiencies such as CVID and XLA. Previous studies have shown that commercial immunoglobulin products,^14^, ^15^, ^16^ increasingly contain SARS-CoV-2-specific Ab, reflecting rising seroprevalence among plasma donors. Our results highlight the importance of accounting for IGRT when interpreting serological data and suggest potential added benefit through passive immunisation in patients unable to mount sufficient endogenous responses.

Cellular immunity analyses in a representative subset revealed robust antigen-specific T cell responses across all study groups. These findings align with prior reports demonstrating durable T cell memory following SARS-CoV-2 vaccination, even in patients with impaired humoural responses.^17^^,^^18^ Both CD4+ and CD8+ memory T cells play a critical role in limiting disease severity and exhibit cross-reactivity across viral variants, including those with substantial antigenic drift from the ancestral strain.^19^, ^20^, ^21^

This study has several strengths. To our knowledge, COVAXID represents one of the longest prospective clinical follow-up studies of SARS-CoV-2 vaccination in immunocompromised patients, with detailed longitudinal sampling, a high patient retention rate, and inclusion of a broad range of immune disorders. These including a group with inherited immunodeficiency expected to remain stable over time; a group of well-controlled individuals living with HIV who demonstrated immune responses comparable to controls; two groups of transplant recipients whose immunosuppressed state improved as the intensity of immunosuppressive therapy was reduced (SOT) or as immunity regained (HSCT); and a group with haematologic malignancy (CLL), known to be highly vulnerable to SARS-CoV-2, in whom ongoing treatment continued to impair immune responses. The integration of matched clinical data, including infection history, vaccination timelines, and IGRTs, enabled a nuanced interpretation of immune trajectories over time. Sample sizes within some subgroups were limited and reflect real-world clinical prevalence rather than formal power calculations. Accordingly, subgroup analyses were exploratory and should be interpreted in the context of longitudinal trends and biological plausibility rather than isolated statistical significance. As such, the analysed cohort represents a real-world clinical population recruited at a tertiary care cancer. Although not designed to be population-representative, all major disease categories and subgroups from the original cohort were retained at three-year follow up. Attrition over time was largely due to logistical and clinical factors inherent to long-term follow-up of immunocompromised patients and should be considered when interpreting longitudinal immune trajectories. Compared with other studies, such as OCTAVE (UK),^22^ COVAXID's prospective design allows for controlled evaluation of temporal dynamics and strengthens causal inference. However, several limitations should be acknowledged. First, it was challenging to distinguish precise time points marking transitions from vaccine-induced to infection-induced “hybrid” immunity. In addition, due to heterogenous and overlapping vaccination, and infection, and where applicable IGRT exposures, formal modelling of Ab waning in relation to time since last antigenic exposure was not readily feasible in this cohort. Furthermore, although anti-nucleocapsid seroconversion remains a useful proxy for infection, it may underestimate cases in some healthy and immunocompromised groups due to transient or absent serological responses.^23^ Conversely, we cannot exclude that IGRT products received by some patients may have contributed to detectable nucleocapsid antibodies, potentially leading to misclassification as infected. At the same time, the absence of documented SARS-CoV-2 infection does not equate to confirmed infection-naïve status in this real-world setting, particularly at later follow-up time points, thereby limiting direct comparisons of vaccine-only immune responses. Multivariable analyses were not performed. Given the heterogeneity of the cohort, limited and uneven subgroup sizes, and strong collinearity between key variables (including vaccination history, SARS-CoV-2 infection, age, and treatment), such models would be prone to overfitting and difficult to interpret robustly. In addition, cumulative antigen exposure is inherently time-dependent and not readily captured in cross-sectional multivariable frameworks. Overall, the findings are likely generalisable to similar patient groups at other sites, irrespective of sex. However, a limitation is that all samples were collected at a single university hospital in Sweden.

The observed maturation of humoural and cellular immunity across the three-year period highlights a key aspect of pandemic preparedness: the ability to build and maintain broad, durable immune protection in high-risk populations. This is particularly relevant as SARS-CoV-2 continues to evolve,^24^^,^^25^ with emerging variants displaying varying degrees of immune escape.^24^^,^^25^ Individuals with reduced baseline immunity or impaired B cell function remain at risk; however, our data suggest that repeated antigenic stimulation, through sustained vaccination coverage and/or natural exposure, can expand neutralisation breadth and improve resilience against divergent variants. Broader and more persistent immune memory may also confer partial protection against future coronaviruses, although the extent of cross-reactivity remains uncertain.

The COVAXID platform provides a valuable framework for evaluating next-generation vaccine strategies in immunocompromised individuals, including bivalent, pan-coronavirus, and mucosal vaccines, which may offer broader protection or more durable protection. Moreover, immunocompromised individuals are also at increased risk from other respiratory pathogens, and lessons learnt from SARS-CoV-2 vaccination may inform broader immunisation strategies, including for influenza and RSV.

In summary, this final three-year analysis of the COVAXID trial cohort delineates that repeated vaccination, natural infection, and IGRT collectively shape long-term SARS-CoV-2 immunity in immunocompromised individuals. The results demonstrate sustained immunological gains across time and patient subgroups, even among those with initially poor responses. These findings emphasise the importance of continued, adaptive vaccination strategies, including risk-tailored booster schedules and access to variant-updated vaccines. Vaccination of elderly and immunocompromised individuals remains important despite detectable Abs, as these populations face faster waning immunity and weaker immune memory. Updated vaccines improve Ab quality and T cell responses, broaden protection against emerging variants, and reduce disease burden, thereby providing essential, multi-layered protection where early on induced immunity may be is insufficient.

## Contributors

PC, PB, OB, LH, SM, PN, GS, AÖ, CIES, KL, MSC, MB, PL, SA, and HGL contributed to conceptualisation of the study, funding acquisition, and discussion of data. PL and SA wrote the original clinical trial protocol. PB, LH, SM, PN, GS, and SA functioned as the primary investigators for each patient study group, recruited study participants and were involved in clinical management during the initial clinical trial and the extended follow-up. OB, AÖ, PL, and JV recruited study participants and were co-responsible for clinical management during the trial. PB, LH, PL, PN, GS, and SA collected and curated clinical data. PC, PB, LH, SM, PN, GS, PL, SA, and HGL were responsible for project administration. PC, MA, DW, AC, and MÅ contributed to project administration by coordinating sample collection and data collection. YG, GB, SMu, and MÅ contributed to investigation through sample analyses. SA and HGL contributed to overall project administration, resources, and supervision of the trial. PC and SA have had access, and verified all underlying clinical data reported in the manuscript. PCperformed, HGL and DW contributed to the visualisation and analysis of all data. YG and OR contributed to the analysis and visualisation of T cell data. HGL wrote the bulk of the manuscript, PC and DW contributed with key input to the manuscript. PB, OB, LH, SM, PN, GS, AÖ, CIES, JV, DW, AC, MÅ, KL, MSC, MB, PL, and SA provided additional comments to the manuscript. All authors have confirmed that they have had full access to the data in the study and have read and approved the manuscript prior to submission.

## Data sharing statement

Relevant data have been submitted to the European Union Drug Regulating Authorities Clinical Trials Database (EudraCT). The full original clinical study protocol is available via the SciLifeLab Data Repository (English version: https://doi.org/10.17044/scilifelab.15059364; Swedish version https://doi.org/10.17044/scilifelab.15059355). Anonymous data displayed in the manuscript can be made available upon request to the corresponding author following publication of the present article. The data, whether displayed in the manuscript or acquired during the clinical trial, can only be made available in a format compliant with local regulatory authorities’ guidelines for handling patient data and in adherence to the policies of the Karolinska University Hospital and Karolinska Institutet.

## Declaration of interests

PB has received honoraria from Takeda and Novartis for educational lectures not directly relevant to this work. SM has received entirely via his institution speaker-fees from Celgene/BMS, Novartis, Janssen, Pfizer, and DNA Prime, research funding from Gilead/Kite, and honoraria as a member and/or head of data safety monitoring boards from Miltenyi and Immunicum not directly relevant to this work. CIES has received financial support from Moderna for work not directly relevant to this work. KL has received financial support from Moderna for work not directly relevant to this work. PL has received grants from Pfizer, MSD, and personal fees from Takeda, MSD, Moderna, and OctaPharma, not directly relevant to this work. MB has served as a consultant and received honoraria from Oxford Immunotech, Gilead, MSD, BMS, Pfizer, and Mabtech, not relevant to this work. SA has received honoraria for lectures from Gilead with payment to Karolinska University Hospital and Karolinska Institutet, participated in advisory boards/consultation for Gilead and Ribocure with waived compensation not directly related to this work, and reports grants from the Swedish Research Council on COVID-19 vaccination. HGL received honoraria from Sanofi and Vycellix for consultation not relevant to this work, served on the UK-CIC Oversight Committee, led the Karolinska Institutet COVID-19 vaccine group, and is on the scientific advisory group for the International Vaccine Institute. All other authors declare no potential or actual conflict of interest to the work presented in this paper.
