# Supplementary material for: Three-year follow-up of the COVAXID trial: real-world assessment of SARS-CoV-2 mRNA vaccine immunogenicity in immunocompromised individuals highlights increasing roles of hybrid and passive immunity
Source: eBioMedicine. 2026 May 8;128:106279. doi: 10.1016/j.ebiom.2026.106279 (PMC13187544; doi:10.1016/j.ebiom.2026.106279)
Supplement: Supplementary Text 1 [file mmc1.docx]

Supplementary Text S1. **Swedish COVID-19 vaccination recommendations during the study period.** National COVID-19 vaccination recommendations in Sweden were issued and regularly updated by the Public Health Agency of Sweden (Folkhälsomyndigheten) in response to evolving epidemiology, vaccine availability, and emerging evidence on immunity and risk groups. In early 2021, vaccination was initiated with a two-dose mRNA (in most cases) primary series, prioritising elderly individuals, residents of long-term care facilities, healthcare workers, and individuals with conditions associated with increased risk of severe COVID-19. Immunocompromised individuals were also identified early as a high-risk group and were therefore prioritised for vaccination once vaccines became available. In this context, current study subjects within the COVAXID clinical trial received their initial vaccination at day 1 and day 21 in line with the original clinical trial study protocol. During the second half of 2021, waning immunity and the emergence of new SARS-CoV-2 variants led to the introduction of booster vaccination. A third vaccine dose was recommended for elderly individuals and risk groups, including immunocompromised patients. For immunocompromised individuals, a three-dose primary vaccination schedule was recommended, reflecting reduced vaccine immunogenicity in these populations. Throughout 2022, booster recommendations were expanded and updated. A third dose was recommended for all adults, while additional booster doses (corresponding to fourth and, in some cases, fifth doses) were advised primarily for elderly individuals and those at increased risk of severe disease. Booster doses were typically recommended at intervals of several months and were often timed ahead of expected waves of increased SARS-CoV-2 transmission, including during periods dominated by Omicron variants. During 2023 and into 2024, as population-level immunity increased due to repeated vaccination and widespread SARS-CoV-2 infection, vaccination recommendations became more targeted. Booster vaccination was mainly recommended for elderly individuals and selected high-risk groups, including immunocompromised patients, generally on an annual basis and preferably using updated (variant-adapted) mRNA vaccines. Consequently, over the three-year study period, participants received varying numbers of vaccine doses in accordance with evolving national recommendations and individual clinical risk assessments. Together with natural SARS-CoV-2 infection, this resulted in heterogeneous cumulative antigen exposure, which is relevant for the interpretation of longitudinal immune responses observed in the present study.
