# Supplementary material for: Three-year follow-up of the COVAXID trial: real-world assessment of SARS-CoV-2 mRNA vaccine immunogenicity in immunocompromised individuals highlights increasing roles of hybrid and passive immunity
Source: eBioMedicine. 2026 May 8;128:106279. doi: 10.1016/j.ebiom.2026.106279 (PMC13187544; doi:10.1016/j.ebiom.2026.106279)

Supplementary Figure S1

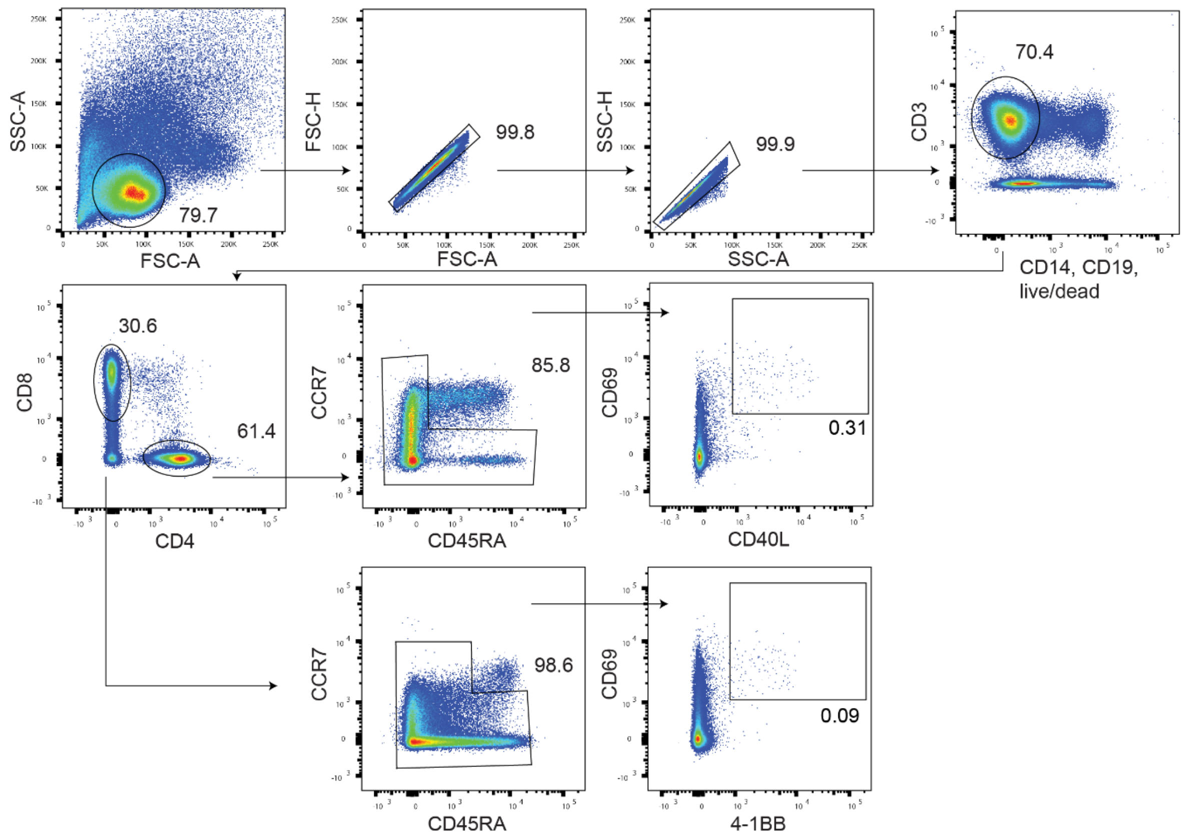

Supplementary Figure S2

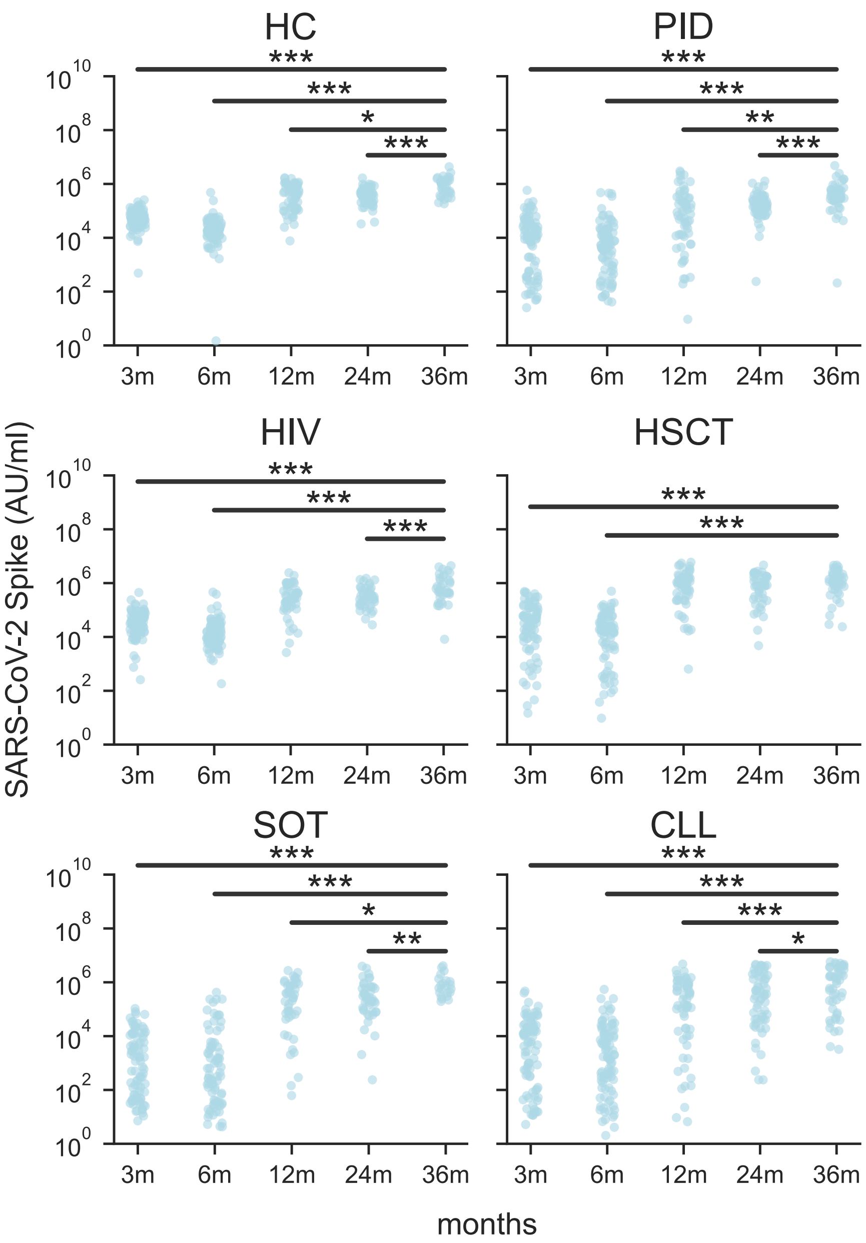

Supplementary Figure S3

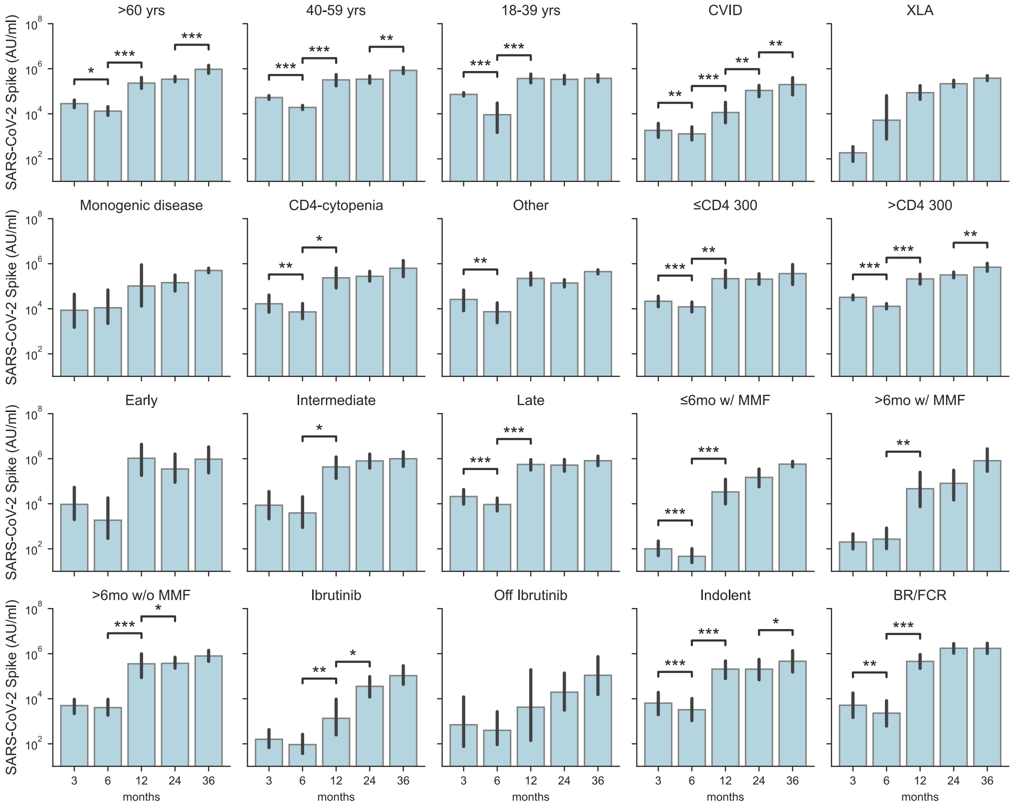

Supplementary Figure S4

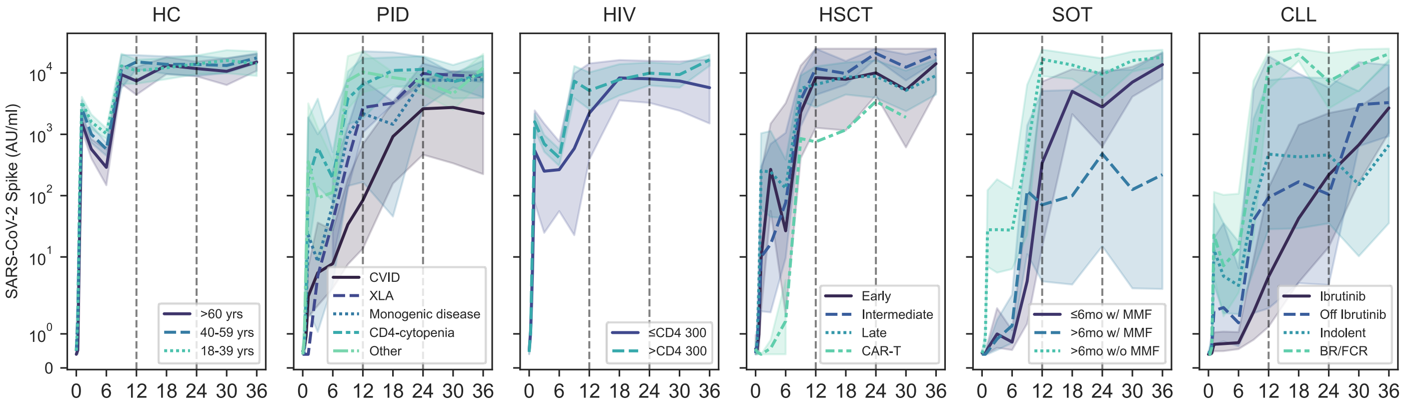

Supplementary Figure S5

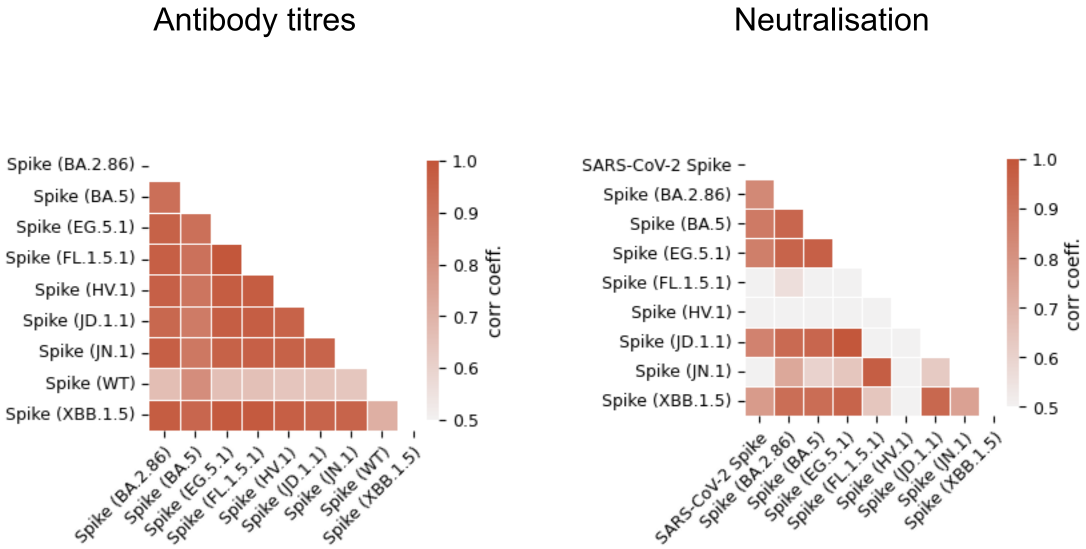

Supplementary Figure S6

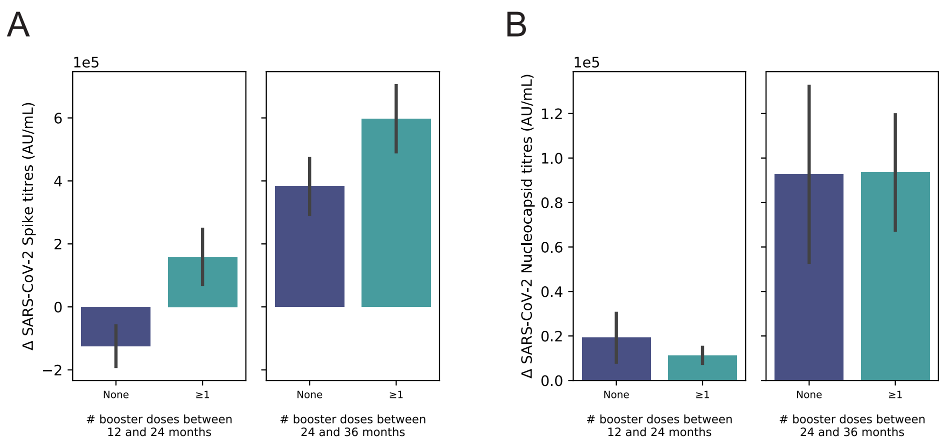

Supplement: Supplementary Figures [file mmc2.pdf]
