# Supplementary material for: Three-year follow-up of the COVAXID trial: real-world assessment of SARS-CoV-2 mRNA vaccine immunogenicity in immunocompromised individuals highlights increasing roles of hybrid and passive immunity
Source: eBioMedicine. 2026 May 8;128:106279. doi: 10.1016/j.ebiom.2026.106279 (PMC13187544; doi:10.1016/j.ebiom.2026.106279)
Supplement: Supplementary Figures Legend [file mmc3.docx]

**Supplementary Figure Legends**

Supplementary Figure S1. **Flow cytometry gating strategy.**Lymphocytes were gated using FSC/SSC, followed by identification of single cells. Live CD3^+^ T cells were gated by excluding dead cells, CD14^+^ cells, and CD19^+^ cells. CD4^+^ and CD8^+^ T cells were identified, and naive cells (defined as CD45RA^+^ and CCR7^+^) were excluded. Antigen-specific CD4^+^ were identified by co-expression of CD69 and CD40L, and antigen-specific CD8^+^ T cells by co-expression of CD69 and 4-1BB.

Supplementary Figure S2. **Dynamics of SARS-CoV-2 antibody titres in the COVAXID cohort.** Data in Figure 1C are presented as dot plots of Spike Wu-Hu.1 Ab titres at 3, 6, 12, 24 and 36 months at the study group level. Statistical tests were performed using the Wilcoxon test with Bonferroni correction for multiple comparisons, using the 36-month time point as reference.

Supplementary Figure S3: **Subgroup-stratified dynamics of SARS-CoV-2 Wu-Hu.1 Ab titres.** Bar plots showing Spike Wu-Hu.1 Ab titres at 3, 6, 12, 24, and 36 months at subgroup levels. With respect to the HSCT group, early/intermediate/late refer to <6 Mo/6-12 Mo/>12 Mo after transplantation at inclusion. Statistical tests were performed using Mann-Whitney, and Bonferroni correction for multiple comparisons. The star annotation (*) indicates statistical significance at a p-value threshold of 0.05 (or ** for p < 0.01, *** for p < 0.001, **** for p < 0.0001).

Supplementary Figure S4: **Dynamics of SARS-CoV-2 Wu-Hu.1 Ab titres.** Assessment of Ab titres of Spike Wu-Hu.1 using the Elecsys platform (geometric mean with 95% CI, shaded range) for each subgroup. The vertical dotted line represents the 1-year and 2-year follow-up sample timepoints. With respect to the HSCT group, early/intermediate/late refer to <6 Mo/6-12 Mo/>12 Mo after transplantation at inclusion.

Supplementary Figure S5. **Correlation matrices assessing SARS-CoV-2 Ab titres and Ab pseudo-neutralising capacity.** Correlation matrices assessing Ab titres and Ab pseudo-neutralising capacity of SARS-CoV-2 subvariants at the 36-month sample timepoint (Spearman rank correlation).

Supplementary Figure S6. **Shift from SARS-CoV-2 vaccine-induced to infection-driven “hybrid”-immunity within the cohort**. (A) Delta SARS-CoV-2 Spike titres and (B) Delta SARS-CoV-2 nucleocapsid titres in relation to none, or one or more, SARS-CoV-2 vaccine booster doses between timepoints 12 to 24 months (left) and 24 to 36 months (right) within (A) and (B), respectively.
